# Electronic Transport in Weyl Semimetals with a Uniform Concentration of Torsional Dislocations

**DOI:** 10.3390/nano12203711

**Published:** 2022-10-21

**Authors:** Daniel Bonilla, Enrique Muñoz

**Affiliations:** Facultad de Física, Pontificia Universidad Católica de Chile, Vicuña Mackenna 4860, Santiago 8331150, Chile

**Keywords:** Weyl semimetals, transport, torsion, dislocations, Kubo relations

## Abstract

In this article, we consider a theoretical model for a type I Weyl semimetal, under the presence of a diluted uniform concentration of torsional dislocations. By means of a mathematical analysis for partial wave scattering (phase-shift) for the T-matrix, we obtain the corresponding retarded and advanced Green’s functions that include the effects of multiple scattering events with the ensemble of randomly distributed dislocations. Combining this analysis with the Kubo formalism, and including vertex corrections, we calculate the electronic conductivity as a function of temperature and concentration of dislocations. We further evaluate our analytical formulas to predict the electrical conductivity of several transition metal monopnictides, i.e., TaAs, TaP, NbAs, and NbP.

## 1. Introduction

Weyl semimetals (WSMs) constitute a remarkable example of three-dimensional, gapless materials with nontrivial topological properties—first proposed theoretically [1,2,3,4,5,6,7] and, more recently, discovered experimentally on TaAs crystals [8].

In a WSM, the band structure possesses an even number of Weyl nodes with linear dispersion, where the conduction and valence bands touch. These nodes are monopolar sources of Berry curvature, and hence are protected from being gapped since their charge (chirality) is a topological invariant [7]. In the vicinity of these nodes, low energy conducting states behave as Weyl fermions, i.e., massless quasi-particles with pseudo-relativistic Dirac linear dispersion [4,5,6,7]. In Weyl fermions, conserved chirality determines the projection of spin over their momentum direction, a condition referred to as “spin-momentum locking”. While Type I WSMs are Lorentz covariant, this symmetry is violated in Type II WSMs, where the Dirac cones are strongly tilted [9].

The presence of Weyl nodes in the bulk spectrum determines the emergence of Fermi arcs [8], the chiral anomaly, and the chiral magnetic effect, among other remarkable properties [9]. Therefore, considerable attention has been paid to understand the electronic transport properties of WSMs [10,11,12]. For instance, there are recent works on charge transport [13] in the presence of spin–orbit coupled impurities [14], electrochemical [15] and nonlinear transport induced by Berry curvature dipoles [16]. Somewhat less explored are the effects of mechanical strain and deformations in WSMs. From a theoretical perspective, it has been proposed that different types of elastic strains can be modeled as gauge fields in WSMs [17,18,19]. In previous works, we have studied the combined effects of a single torsional dislocation and an external magnetic field on the electronic [20,21] and thermoelectric [20,22] transport properties of WSMs, using the Landauer ballistic formalism in combination with a mathematical analysis for the quantum mechanical scattering cross-sections [23].

In this work, we extend our previous analysis to study the case of a diluted, uniform concentration of torsional dislocations and its effects on the electrical conductivity of type I WSMs. In contrast to our former studies [20,21,22], here we employ the Kubo linear-response formalism at finite temperatures. This requires explicitly calculating the retarded and advanced Green´s functions for the system, including the multiple scattering events due to the random distribution of dislocation defects in the form of a disorder-averaged self-energy term. For this purpose, we first analyze the scattering phase shift arising from a single torsional dislocation, and then we obtain the corresponding (retarded and advanced) Green’s function in terms of the T-matrix elements by solving analytically the Lippmann–Schwinger equation. We further extend this analysis, by incorporating the effect of a random distribution of such dislocations, with a concentration $n_{d}$, in the form of a disorder-averaged self-energy into the corresponding Dyson’s equation. Finally, we analyze the correction due to the scattering vertex, and by including this additional contribution, we calculate the electrical conductivity from the Kubo formula, as a function of temperature and concentration of dislocations. We present explicit evaluations of our analytical expressions for the electrical conductivity as a function of temperature and concentration of dislocations $n_{d}$, for several materials in the family of transition metals’ monopnictides, i.e., TaAs, TaP, NbAs and NbP, where the corresponding microscopic parameters, estimated by ab initio methods, were reported in the literature [24,25,26].

## 2. Scattering by a Single Dislocation

**Figure 1:**
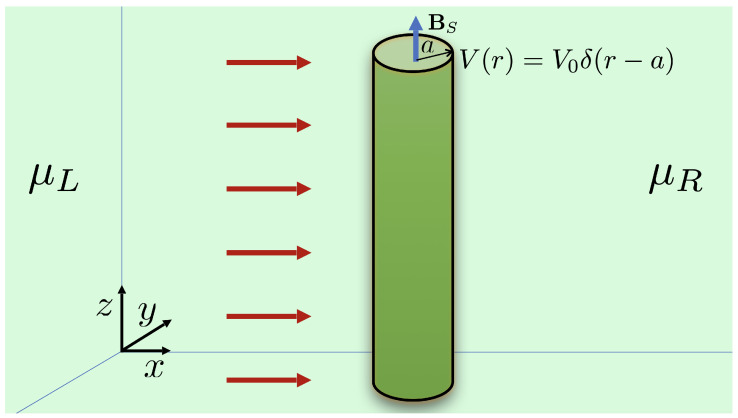
Pictorial description of the scattering of free incident Weyl fermions coming from a left reservoir by a single cylindrical dislocation defect.

As a continuum model for a type I WSM under the presence of a single dislocation defect, as depicted in Figure 1, we consider the Hamiltonian [22]

(1) $${\hat{H}}^{\xi }=\xi \hbar v_{F}\sigma \cdot (p+eA^{\xi })+\sigma_{0}V_{0}\delta \left(r-a\right)\equiv {\hat{H}}_{0}^{\xi }+{\hat{H}}_{1}^{\xi },$$

where

(2) $$\begin{array}{cc} {\hat{H}}_{0}^{\xi } & =\xi v_{F}\sigma \cdot p, \end{array}$$

(3) $$\begin{array}{cc} {\hat{H}}_{1}^{\xi } & =\xi ev_{F}\left(\sigma \cdot \hat{\phi }\right)\frac{1}{2}B^{\xi }r\Theta \left(a-r\right)+V_{0}\delta \left(r-a\right)\sigma_{0}. \end{array}$$

Here, ξ=± labels each of the Weyl nodes located at $K_{\pm }=\pm \;b/2$. The expression in Equation (2) is the free-particle Hamiltonian, whereas the expression in Equation (3) represents the interaction with the dislocation, where torsional strain is described as a pseudo-magnetic field inside the cylinder [21,22,23], as well as the lattice mismatch effect at the boundary of the dislocation, modeled as a repulsive delta barrier on its surface [22].

The “free” spinor eigenfunctions for the defect-free reference system satisfy

(4) $${\hat{H}}_{0}^{\xi }|\Phi_{k,\lambda }\rangle =E_{\lambda ,k}^{\left(0,\xi \right)}|\Phi_{k,\lambda }\rangle ,$$

where the energy spectrum is given by

(5) $$E_{\lambda ,k}^{\left(0,\xi \right)}=\lambda \xi \hbar v_{F}\left|k\right|,$$

and λ= ±1 is the band (helicity) index. When projected onto coordinate space, these spinor eigenfunctions have the explicit form

(6) $$\Phi_{\lambda ,k}\left(r\right)=\sqrt{\frac{\left|k\right|+\lambda k_{z}}{2|k|}}\left(\begin{array}{c} 1\\ \frac{\lambda |k|-k_{z}}{k_{x}-ik_{y}} \end{array}\right)e^{ik\cdot r},$$

and constitute an orthonormal basis for the Hilbert space.

If we now consider the (elastic) scattering effects induced by the torsional dislocation modeled by Equation (3), we need to look for the eigenvectors $|\Psi_{\lambda ,k}\rangle$ of the total Hamiltonian in Equation (1) with the same energy as in Equation (5). The answer is provided by the solution to the well-known Lippmann–Schwinger equation

(7) $$|\Psi_{k,\lambda }\rangle =|\Phi_{k,\lambda }\rangle +{\hat{G}}_{R,0}^{\xi }\left(E\right){\hat{H}}_{1}^{\xi }|\Psi_{k,\lambda }\rangle ,$$

where the free Green’s function can be expressed in a coordinate-independent representation form via the *resolvent*,

(8) $${\hat{G}}_{R/A,0}^{\xi }\left(E\right)={\left[E-{\hat{H}}_{0}^{\xi }\pm i\eta^{+}\right]}^{-1}.$$

Here, the index R/A stands for retarded and advanced, respectively. As shown in detail in Appendix A, in the coordinate representation, the corresponding free Green’s function is given by the explicit matrix form $G_{R,0}^{\xi }\left(r,r^{\prime };k\right)=\delta \left(z-z^{\prime }\right)G_{R,0}^{\xi }\left(x,x^{\prime };k\right)$, where r=(x,z) and

(9) $$\begin{array}{c} G_{R,0}^{\xi }\left(x,x^{\prime };k\right)=-\frac{\lambda \xi ik}{4\hbar v_{F}}\left[\begin{array}{cc} H_{0}^{\left(1\right)}\left(k|x-x^{\prime }|\right) & i\lambda e^{-i\varphi }H_{1}^{\left(1\right)}\left(k|x-x^{\prime }|\right)\\ i\lambda e^{i\varphi }H_{1}^{\left(1\right)}\left(k|x-x^{\prime }|\right) & H_{0}^{\left(1\right)}\left(k|x-x^{\prime }|\right) \end{array}\right]. \end{array}$$

Here, $H_{0}^{\left(1\right)}\left(z\right)$ and $H_{1}^{\left(1\right)}\left(z\right)$ are the Hankel functions, and x=(x,y) is the position vector on any plane perpendicular to the cylinder’s axis.

For the scattering analysis, we need the retarded resolvent for the full Hamiltonian, which is defined as the solution to the equation

(10) $$\left(E+i\eta^{+}-{\hat{H}}^{\xi }\right){\hat{G}}_{R}^{\xi }\left(E\right)=\hat{I}.$$

Combining Equation (10) with Equation (8), we readily obtain

(11) $$\begin{array}{cc} {\hat{G}}_{R}^{\xi }\left(E\right) & ={\hat{G}}_{R,0}^{\xi }\left(E\right)+{\hat{G}}_{R,0}^{\xi }\left(E\right){\hat{H}}_{1}^{\xi }{\hat{G}}_{R}^{\xi }\left(E\right)\\ \; & ={\hat{G}}_{R,0}^{\xi }\left(E\right)+{\hat{G}}_{R,0}^{\xi }\left(E\right){\hat{T}}^{\xi }\left(E\right){\hat{G}}_{R,0}^{\xi }\left(E\right), \end{array}$$

where we introduced the standard definition of the T-matrix operator ${\hat{T}}^{\xi }\left(E\right)$ that can be formally expressed in closed form by

(12) $$\begin{array}{cc} {\hat{T}}^{\xi }\left(E\right) & ={\hat{H}}_{1}^{\xi }+{\hat{H}}_{1}^{\xi }{\hat{G}}_{R,0}^{\xi }\left(E\right){\hat{T}}^{\xi }\left(E\right)\\ \; & ={\hat{H}}_{1}^{\xi }{\left(\hat{I}-{\hat{G}}_{R,0}^{\xi }\left(E\right){\hat{H}}_{1}^{\xi }\right)}^{-1}. \end{array}$$

Using this definition, along with the property ${\hat{H}}_{1}^{\xi }|\Psi_{k,\lambda }\rangle ={\hat{T}}^{\xi }|\Phi_{k,\lambda }\rangle$, we obtain the Lippmann–Schwinger Equation (7) in the coordinate representation

(13) $$\Psi_{k,\lambda }\left(r\right)=\Phi_{k,\lambda }\left(r\right)\;+\int d^{3}r^{\prime }\int d^{3}r^{\prime \prime }\langle r|{\hat{G}}_{R,0}^{\xi }\left(E\right)|r^{\prime }\rangle \langle r^{\prime }\left|{\hat{T}}^{\xi }\left(E\right)\right|r^{\prime \prime }\rangle \Phi_{k,\lambda }\left(r^{\prime \prime }\right).$$

As shown in detail in Appendix B, by considering the asymptotic behavior of the Hankel functions, $H_{\nu }^{\left(1\right)}\left(x\right)∼\sqrt{\frac{2}{\pi x}}e^{i\left(x-\frac{\nu \pi }{2}-\frac{\pi }{4}\right)}$ (for x→∞), Equation (13) can be reduced to the *x*–*y* plane and takes the explicit asymptotic expression

(14) $$\Psi_{k_{\|},\lambda }\left(x\right)∼\frac{1}{\sqrt{2}}\left(\begin{array}{c} 1\\ \lambda \end{array}\right)e^{ikx}-\frac{\lambda \xi }{2\hbar v_{F}}\sqrt{\frac{ik}{\pi }}T_{k_{\|}^{\prime }k_{\|}}^{\left(\lambda ,\xi \right)}\left(\begin{array}{c} 1\\ \lambda e^{i\phi } \end{array}\right)\frac{e^{ikr}}{\sqrt{r}},$$

where, as we explain in the Appendix, the particles have only momenta perpendicular to the defect’s axis, i.e., $k_{\|}=\left(k_{x},k_{y}\right)$. Comparing this last result with our previous reported expression for the scattering amplitude [27],

(15) $$\left[\begin{array}{c} f_{1}\left(\phi \right)\\ f_{2}\left(\phi \right) \end{array}\right]=\frac{e^{-\frac{i\pi }{4}}}{\sqrt{4\pi k}}\sum_{m=-\infty }^{\infty }\left[\begin{array}{c} e^{im\phi }\\ \lambda e^{i(m+1)\phi } \end{array}\right]\left(e^{2i\delta_{m}}-1\right),$$

we identify $T_{k^{\prime }k}^{\left(\lambda ,\xi \right)}=-2\lambda \xi \hbar v_{F}\sqrt{\pi /ik}f_{1}\left(\phi \right)$. Therefore, we arrived at an explicit analytical expression for the T-matrix elements in terms of the phase shift $\delta_{m}\left(k\right)$ for each angular momentum channel m∈Z

(16) $$T_{k_{\|}^{\prime }k_{\|}}^{\left(\lambda ,\xi \right)}=-\frac{2\lambda \xi \hbar v_{F}}{k}\sum_{m=-\infty }^{\infty }e^{i\delta_{m}\left(k\right)}\sin\delta_{m}\left(k\right)e^{im\phi },$$

where ϕ is the angle between $k_{\|}$ and $k_{\|}^{\prime }$, and the analytical expression for the phase shift is given in Appendix B by Equation (A27).

**Figure 2:**
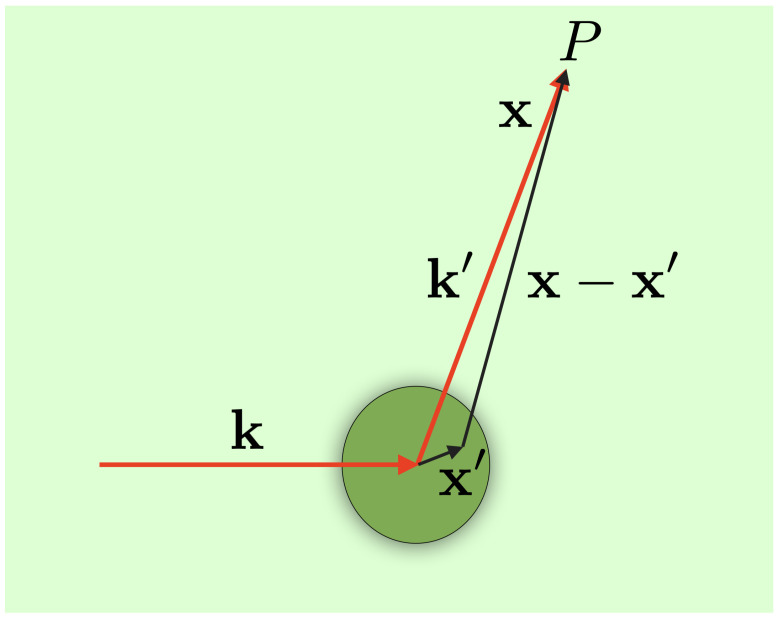
Pictorial description of the scattering event on a plane perpendicular to the cylindrical defect axis.

## 3. Scattering by a Uniform Concentration of Dislocations

**Figure 3:**
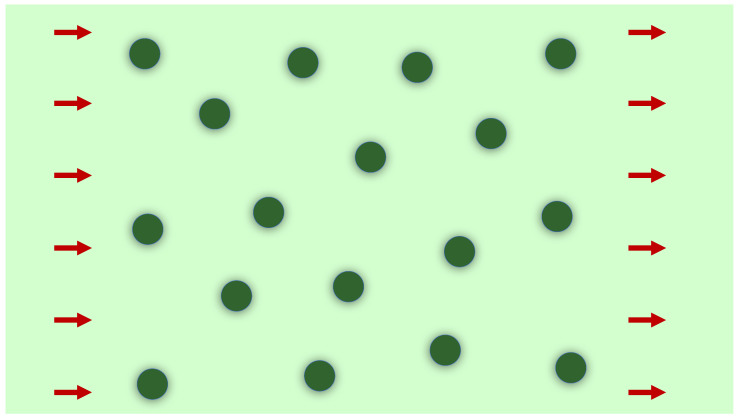
Random distribution of torsional dislocations seen from a plane perpendicular to the cylinder axis.

Let us now consider a uniform concentration $n_{d}=N_{d}/A$ (per unit transverse surface) of identical cylindrical dislocations, as depicted in Figure 3, represented by the density function

(17) $$\rho \left(x\right)=\sum_{j=1}^{N_{d}}\delta \left(x-X_{j}\right),$$

where $X_{j}$ is the position of the $j^{th}$-dislocation’s axis. The Fourier transform of this density function is thus given by the expression

(18) $$\tilde{\rho }\left(k_{\|}\right)=\int d^{2}xe^{-ik_{\|}\cdot x}\rho \left(x\right)=\sum_{j=1}^{N_{d}}e^{-ik_{\|}\cdot X_{j}}.$$

The operator that plays the role of a scattering potential for this distribution of dislocation defects is

(19) $$V\left(x\right)=\int d^{2}x^{\prime }\rho \left(x^{\prime }\right)H_{1}^{\xi }\left(x-x^{\prime }\right)=\sum_{j=1}^{N_{d}}H_{1}^{\xi }\left(x-X_{j}\right),$$

where $H_{1}^{\xi }$ was defined in Equation (3) as the contribution from a single dislocation. The matrix elements of the scattering operator Equation (19) in the free spinor basis defined by Equation (4) are

(20) $$\begin{array}{c} \langle \Phi_{k_{\|},\lambda }|V\left(x\right)|\Phi_{k_{\|}^{\prime },\lambda^{\prime }}\rangle ={\left[\tilde{V}\left(k_{\|}-k_{\|}^{\prime }\right)\right]}_{\lambda \lambda^{\prime }}, \end{array}$$

where $\tilde{V}\left(k_{\|}\right)$ is the Fourier transform of V(x):

(21) $$\begin{array}{cc} \tilde{V}\left(k_{\|}\right) & =\int_{R^{2}}d^{2}xe^{-ik_{\|}\cdot x}V\left(x\right)=\sum_{j=1}^{N_{d}}\int_{R^{2}}d^{2}xe^{-ik_{\|}\cdot x}H_{1}^{\xi }\left(x-X_{j}\right)\\ \; & ={\tilde{H}}_{1}^{\xi }\left(k_{\|}\right)\tilde{\rho }\left(k_{\|}\right). \end{array}$$

Then, the matrix elements of the potential in Equation (20) become

(22) $${\left[\tilde{V}\left(k_{\|}\right)\right]}_{\lambda \lambda^{\prime }}={\left[{\tilde{H}}_{1}^{\xi }\left(k_{\|}\right)\right]}_{\lambda \lambda^{\prime }}\tilde{\rho }\left(k_{\|}\right).$$

Let us also introduce the configurational average of a function $f(X_{j})$ over the statistical distribution of dislocations as

(23) $$\langle f\rangle =\int_{R^{2}}d^{2}X_{j}\;\;P\left(X_{j}\right)f\left(X_{j}\right),$$

where $P(X_{j})$ is the normalized distribution function for the defects in the sample. In particular, for a uniform distribution, we have $P(X_{j})=1/A$, where *A* is the area of the plane normal to each cylinder’s axis. Now, the full retarded Green’s function under the presence of several dislocations represented by the operator $\hat{V}$ given in Equation (19) satisfies the equation

(24) $${\hat{G}}_{R}^{\xi }\left(E\right)={\hat{G}}_{R,0}^{\xi }\left(E\right)+{\hat{G}}_{R,0}^{\xi }\left(E\right)\hat{V}{\hat{G}}_{R}^{\xi }\left(E\right).$$

The configurational average, as defined in Equation (23), of the full Green’s function in this last equation can be written as

(25) $$\langle {\hat{G}}_{R}^{\xi }\left(E\right)\rangle ={\hat{G}}_{R,0}^{\xi }\left(E\right)+{\hat{G}}_{R,0}^{\xi }\left(E\right)\Sigma_{R}^{\lambda ,\xi }\left(E\right)\langle {\hat{G}}_{R}^{\xi }\left(E\right)\rangle .$$

This is the Dyson’s equation with the retarded self-energy $\Sigma_{R}^{\lambda ,\xi }\left(E\right)$ that can be explicitly solved to yield

(26) $$\langle G_{R}^{\lambda ,\xi }\left(k_{\|}\right)\rangle =\frac{1}{E-\lambda \xi \hbar v_{F}\left|k_{\|}\right|-\Sigma_{R}^{\lambda ,\xi }\left(k_{\|}\right)}.$$

The effect of the statistical distribution of dislocations’ is entirely determined by the function $\tilde{\rho }\left(k_{\|}\right)$. In the perturbative expansion of the full Green’s function, we encounter *n*th-products of the form $\tilde{\rho }\left(k_{1}\right)\tilde{\rho }\left(k_{2}\right)\cdots \tilde{\rho }\left(k_{n}\right)$. The configurational average of a single factor is given by

(27) $$\begin{array}{cc} \langle \tilde{\rho }\left(k_{\|}\right)\rangle & =\langle \sum_{j=1}^{N_{d}}e^{-ik_{\|}\cdot X_{j}}\rangle =\sum_{j=1}^{N_{d}}\int_{R^{2}}d^{2}X_{j}\;\frac{1}{A}\;e^{-ik_{\|}\cdot X_{j}}=\frac{N_{d}}{A}{\left(2\pi \right)}^{2}\delta^{\left(2\right)}\left(k_{\|}\right), \end{array}$$

**Figure 4:**
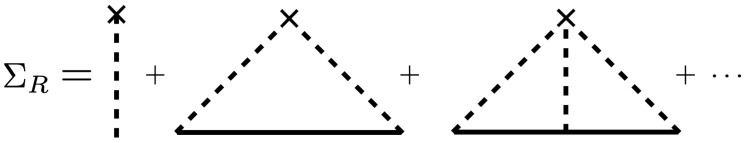
Diagrams contributing to the retarded self-energy $\Sigma_{R}$. The solid line corresponds to the free retarded Green’s function, the dashed line the scattering perturbation $H_{1}$, and the × a factor of $n_{d}$.

Similarly, for the product of two factors, we obtain

(28) $$\begin{array}{cc} \langle \tilde{\rho }\left(k_{\|}^{1}\right)\tilde{\rho }\left(k_{\|}^{2}\right)\rangle & =\langle \sum_{j=1}^{N_{d}}\sum_{l=1}^{N_{d}}e^{-ik_{\|}^{1}\cdot X_{j}-ik_{\|}^{2}\cdot X_{l}}\rangle =\langle \sum_{j=l}e^{-i\left(k_{\|}^{1}+k_{\|}^{2}\right)\cdot X_{j}}+\sum_{j\neq l}e^{-ik_{\|}^{1}\cdot X_{j}-ik_{\|}^{2}\cdot X_{l}}\rangle \\ \; & =\frac{N_{d}}{A}{\left(2\pi \right)}^{2}\delta^{\left(2\right)}\left(k_{\|}^{1}+k_{\|}^{2}\right)+\frac{N_{d}\left(N_{d}-1\right)}{A^{2}}{\left(2\pi \right)}^{4}\delta^{\left(2\right)}\left(k_{\|}^{1}\right)\delta^{\left(2\right)}\left(k_{\|}^{2}\right), \end{array}$$

and we have a similar behavior for higher order products. Now, notice that, for $N_{d}\gg 1$, we have $N_{d}\left(N_{d}-1\right)\approx N_{d}^{2}$, $N_{d}\left(N_{d}-1\right)\left(N_{d}-3\right)\approx N_{d}^{3}$ and so on. We define the concentration of defects, i.e., the number of dislocations per unit of area perpendicular to the cylinder’s axis as $n_{d}=N_{d}/A$. As discussed in standard references [28,29], for small concentrations $n_{d}\ll 1$, the scaling discussed before ensures that the total Green’s function in Equation (25) can be calculated accurately by the sequence of diagrams for the retarded self-energy in momentum space as given in Figure 4, an approach well known as the non-crossing approximation (NCA). This series of diagrams corresponds to the configurational average of the *T*-matrix over the random distribution of dislocations after Equation (23)

(29) $$\Sigma_{R}^{\lambda ,\xi }\left(E\right)=\langle {\hat{T}}^{\xi }\left(E\right)\rangle =n_{d}T_{k_{\|}k_{\|}}^{\left(\lambda ,\xi \right)}.$$

Using the expression in Equation (16) for the *T*-matrix elements, for $k_{\|}=k_{\|}^{\prime }$ that implies ϕ=0, we have that the real part of the self-energy

(30) ReΣRλ,ξ(k‖)=−2λξndℏvFk∑m=−∞∞cosδm(λ,ξ)(k)sinδm(λ,ξ)(k)

contains an infinite sum over highly oscillatory terms that converges to zero. Therefore, no contribution arises from the real part of the self-energy. The imaginary part, on the other hand, leads to the definition of the relaxation time,

(31) 1τ(λ,ξ)(k)=−2λξℏndImTk‖k‖(λ,ξ).

### 3.1. Electrical Conductivity in the Linear-Response Regime

In order to arrive at the definition of the electrical conductivity in the linear response regime, let us first consider a single Fourier mode for an external electric field E, in the gauge

(32) $$E=-\frac{\partial }{\partial t}A\left(r,t\right),$$

where $A\left(r,t\right)=A\left(r,\omega \right)e^{-i\omega t}$ is the vector potential. Then, E=iωA. In the linear response formalism, the current is given by the expression

(33) $$j_{\alpha }\left(r,\omega \right)=\int d^{3}r^{\prime }\;\sigma_{\alpha \beta }\left(r,r^{\prime };\omega \right)E_{\beta }\left(r^{\prime }.\omega \right),$$

where the conductivity tensor is defined by

(34) $$\sigma_{\alpha \beta }\left(r,r^{\prime };\omega \right)=\frac{1}{i\omega }K_{\alpha \beta }\left(r,r^{\prime };\omega \right).$$

In the Kubo formalism, the tensor $K_{\alpha \beta }$ is defined in terms of the retarded current-current correlator as follows:

(35) $$K_{\alpha \beta }\left(r,t;r^{\prime },t^{\prime }\right)=i\hbar^{-1}\theta \left(t-t^{\prime }\right)\mathrm{Tr}\left\{\hat{\rho }\left[{\hat{j}}_{\alpha }\left(r,t\right),{\hat{j}}_{\beta }\left(r^{\prime },t^{\prime }\right)\right]\right\},$$

where $\hat{\rho }$ is the statistical density matrix operator. As shown in detail in Appendix D, the Fourier transform of this tensor to the frequency domain is given by

(36) $$K_{\alpha \beta }^{\xi }\left(r,r^{\prime };\omega \right)=e^{2}v_{F}^{2}\int_{-\infty }^{\infty }\frac{dE^{\prime }}{2\pi }\int_{-\infty }^{\infty }\frac{dE}{2\pi }\frac{f_{0}\left(E^{\prime }\right)-f_{0}\left(E\right)}{\hbar \omega +E-E^{\prime }+i\eta^{+}}\mathrm{Tr}\left[\sigma_{\alpha }{𝓐}^{\xi }\left(r,r^{\prime };E^{\prime }\right)\sigma_{\beta }{𝓐}^{\xi }\left(r^{\prime },r;E\right)\right].$$

Here, $f_{0}\left(E\right)={\left[e^{(E-\mu )/kT}+1\right]}^{-1}$ is the Fermi distribution, and we introduced the (disorder-averaged) spectral function

(37) $$\begin{array}{c} A^{\lambda ,\xi }\left(k\right)=i\left[\langle G_{R}^{\lambda ,\xi }\left(k_{\|}\right)\rangle -\langle G_{A}^{\lambda ,\xi }\left(k_{\|}\right)\rangle \right]=\frac{2\left(\frac{\hbar }{2\tau^{\left(\lambda ,\xi \right)}\left(k\right)}\right)}{{\left(E-E_{k}^{\lambda ,\xi }\right)}^{2}+{\left(\frac{\hbar }{2\tau^{\left(\lambda ,\xi \right)}\left(k\right)}\right)}^{2}} \end{array}$$

that clearly represents a Lorentzian distribution whose spectral width is defined by the inverse of the relaxation time (see Appendix C for the details). After some algebraic manipulations, we obtain the conductivity tensor at finite frequency and temperature

(38) $$\begin{array}{cc} ℜ\;\sigma_{\alpha \beta }^{\xi }\left(r,r^{\prime };\omega \right)= & -\frac{e^{2}\hbar v_{F}^{2}}{2\pi }\int_{-\infty }^{\infty }dE\left\{\frac{f_{0}\left(E+\hbar \omega \right)-f_{0}\left(E\right)}{\hbar \omega }\right\}\\ \; & \;\;\times \mathrm{Tr}[\sigma_{\alpha }{𝓐}^{\xi }\left(r,r^{\prime };E+\hbar \omega \right)\sigma_{\beta }{𝓐}^{\xi }\left(r^{\prime },r;E\right)]. \end{array}$$

Using the coordinates representation of the spectral function given in Appendix C, after Equation (A33), we can read off the Fourier transform to momentum space of the conductivity

$$\begin{array}{cc} \sigma_{\alpha \beta }^{\xi }\left(q;\omega \right)= & -\frac{e^{2}\hbar v_{F}^{2}}{2\pi }\int \frac{d^{3}k}{{\left(2\pi \right)}^{3}}\int_{-\infty }^{\infty }dE\left\{\frac{f_{0}\left(E+\hbar \omega \right)-f_{0}\left(E\right)}{\hbar \omega }\right\}\\ \; & \;\;\times \sum_{\lambda ,\lambda^{\prime }}\mathrm{Tr}\left\{\sigma_{\alpha }\left(\sigma_{0}+\lambda \frac{\sigma \cdot (k_{\|}+q)}{|k_{\|}+q|}\right)\sigma_{\beta }\left(\sigma_{0}+\lambda^{\prime }\frac{\sigma \cdot k_{\|}}{|k_{\|}|}\right)\right\} \end{array}$$

(39) $$\begin{array}{c} \;\;\times A^{\lambda ,\xi }\left(\right|k_{\|}+q|;E+\hbar \omega )A^{\lambda^{\prime },\xi }\left(\right|k_{\|}\left|;E\right). \end{array}$$

We are interested in the DC conductivity, so we take the limit q→0 first and then the limit ω→0. After a long calculation (details in the Appendix D), the result is

(40) $$\begin{array}{cc} \sigma_{\alpha \beta }^{\left(\lambda ,\xi \right)}\left(T\right) & =\delta_{\alpha \beta }\frac{e^{2}\hbar v_{F}^{2}}{\pi^{3}}\int_{0}^{\infty }dk\;\;\int_{-\infty }^{\infty }dE\;\;\left(-\frac{\partial f_{0}\left(E\right)}{\partial E}\right)\langle G_{R}^{\lambda ,\xi }\left(k_{\|}\right)\rangle \langle G_{A}^{\lambda ,\xi }\left(k_{\|}\right)\rangle k_{\|}\cdot k_{\|}. \end{array}$$

### 3.2. Vertex Corrections

**Figure 5:**
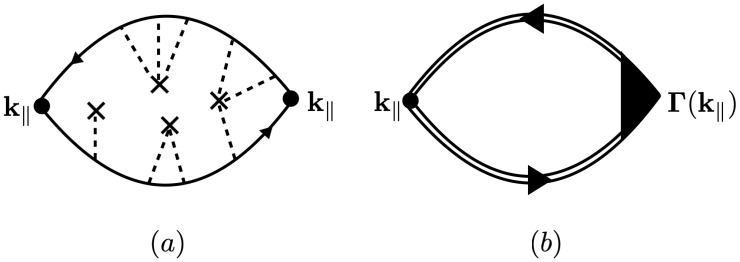
(**a**) A typical diagram contributing to the conductivity in Equation (40), involving the configurational average of the two internal GF with cross-links between them. The upper line corresponds to the retarded GF and the lower to the advanced GF; (**b**) diagrammatic representation of the two complete averaged GF (double lines) corresponding to the sum of all diagrams of the kind in (**a**) with the vertex correction $\Gamma (k_{\|})$.

The self-energy contribution modifies the definition of the retarded and advanced Green´s functions in Equation (40), as depicted by the double lines in Figure 5b. However, there are also scattering processes involving links between the two internal Green function lines, as depicted in Figure 5a. When considering such diagrams with cross-links, as in Figure 5a, we must include the vertex correction as depicted in Figure 5b.

**Figure 6:**
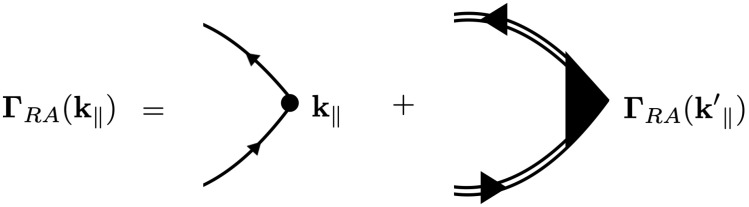
The Bethe–Salpeter integral equation for the vertex function $\Gamma_{RA}\left(k_{\|}\right)$.

Taking into account the vertex correction, the conductivity becomes

(41) $$\begin{array}{cc} \sigma_{\alpha \beta }^{\left(\lambda ,\xi \right)}\left(T\right) & =\delta_{\alpha \beta }\frac{e^{2}\hbar v_{F}^{2}}{\pi^{3}}\int_{0}^{\infty }dk\;\;\int_{-\infty }^{\infty }dE\;\;\left(-\frac{\partial f_{0}\left(E\right)}{\partial E}\right)\langle G_{R}^{\lambda ,\xi }\left(k_{\|}\right)\rangle \langle G_{A}^{\lambda ,\xi }\left(k_{\|}\right)\rangle k_{\|}\cdot \Gamma_{RA}\left(k_{\|},E\right), \end{array}$$

where the vertex function $\Gamma_{RA}\left(k_{\|},E\right)$ is given as the solution to the *Bethe-Salpeter equation* as depicted in Figure 6. Then, we have

(42) $$\begin{array}{c} \Gamma_{RA}\left(k_{\|},E\right)=k_{\|}+n_{d}\int \frac{d^{2}k^{\prime }}{{\left(2\pi \right)}^{2}}\langle G_{R}^{\lambda ,\xi }\left(k_{\|}^{\prime }\right)\rangle \langle G_{A}^{\lambda ,\xi }\left(k_{\|}^{\prime }\right)\rangle {\left|T_{k_{\|}^{\prime }k_{\|}}^{\left(\lambda ,\xi \right)}\right|}^{2}\Gamma_{RA}\left(k_{\|}^{\prime },E\right). \end{array}$$

The iterative solution of Equation (42) for $\Gamma_{RA}\left(k_{\|},E\right)$ shows that the vertex function must be of the form

(43) $$\Gamma_{RA}\left(k_{\|},E\right)=\gamma \left(k_{\|},E\right)k_{\|}.$$

Then, we obtain a secular integral equation for the scalar function $\gamma (k_{\|},E)$ that in the low concentration limit becomes

(44) $$\begin{array}{c} \gamma \left(k_{\|},E\right)=1+n_{d}\frac{2\pi }{\hbar }\int \frac{d^{2}k^{\prime }}{{\left(2\pi \right)}^{2}}\tau^{\left(\lambda ,\xi \right)}\left(k^{\prime }\right){\left|T_{k_{\|}^{\prime }k_{\|}}^{\left(\lambda ,\xi \right)}\right|}^{2}\delta \left(E-\lambda \xi \hbar v_{F}k^{\prime }\right)\;\;\gamma \left(k_{\|}^{\prime },E\right)\frac{k_{\|}\cdot k_{\|}^{\prime }}{k^{2}}. \end{array}$$

In the limit of low concentrations, we use the result in Appendix D, Equation (A46), to obtain

(45) $$\begin{array}{c} \sigma_{\alpha \alpha }^{\left(\lambda ,\xi \right)}\left(T\right)=\frac{2e^{2}v_{F}^{2}}{\pi^{2}}\int_{0}^{\infty }dk\;k^{2}\;{\left(-\frac{\partial f_{0}\left(E\right)}{\partial E}\right)}_{E=\lambda \xi \hbar v_{F}k}\tau^{\left(\lambda ,\xi \right)}\left(k\right)\;\;\gamma \left(k_{\|},\lambda \xi \hbar v_{F}k\right). \end{array}$$

At low temperatures, an exact solution is possible since the derivative of the Fermi distribution takes a compact support at the Fermi energy. Therefore, we can evaluate γ(k) and $\tau^{\left(\lambda ,\xi \right)}\left(k\right)$ at the Fermi momentum $k_{F}^{\xi }$, to obtain

(46) $$\gamma \left(k_{F}^{\xi }\right)=\frac{\tau_{1}^{\left(\lambda ,\xi \right)}\left(k_{F}^{\xi }\right)}{\tau_{1}^{\left(\lambda ,\xi \right)}\left(k_{F}^{\xi }\right)-\tau^{\left(\lambda ,\xi \right)}\left(k_{F}^{\xi }\right)},$$

where we defined (for $\cos\phi^{\prime }=k_{\|}\cdot k_{\|}^{\prime }/k^{2}$)

(47) $$\frac{1}{\tau_{1}^{\left(\lambda ,\xi \right)}\left(k_{F}^{\xi }\right)}=n_{d}\frac{2\pi }{\hbar }\int \frac{d^{2}k^{\prime }}{{\left(2\pi \right)}^{2}}{\left|T_{k_{\|}^{\prime }k_{\|}}^{\left(\lambda ,\xi \right)}\right|}^{2}\cos\phi^{\prime }\;\;\delta \left(\hbar v_{F}k_{F}^{\xi }-\hbar v_{F}k^{\prime }\right).$$

After the substitution of $\gamma (k_{\|},E)$ from Equation (46) into Equation (45), we finally obtain a closed analytical expression for the bulk electrical conductivity, as a function of temperature and concentration of dislocations $n_{d}$

(48) $$\begin{array}{cc} \sigma_{\alpha \alpha }^{\left(\lambda ,\xi \right)}\left(T\right) & =\frac{2e^{2}v_{F}^{2}}{\pi^{2}k_{B}T}\tau_{\mathrm{tr}}^{\left(\lambda ,\xi \right)}\left(k_{F}^{\xi }\right)\int_{0}^{\infty }dk\;k^{2}\;f_{0}\left(E_{k_{\|}}^{\lambda ,\xi }\right)\left[1-f_{0}\left(E_{k_{\|}}^{\lambda ,\xi }\right)\right]\\ \; & =-\frac{4}{\pi^{2}v_{F}}\left(\frac{e^{2}}{\hbar }\right){\left(\frac{k_{B}T}{\hbar }\right)}^{2}\tau_{\mathrm{tr}}^{\left(\lambda ,\xi \right)}\left(k_{F}^{\xi }\right)\;{\mathrm{Li}}_{2}\left(-e^{\frac{\hbar v_{F}k_{F}}{k_{B}T}}\right), \end{array}$$

where ${\mathrm{Li}}_{2}\left(x\right)$ is the polylogarithm of order 2. Here, the total *transport relaxation time* is defined by

(49) $$\begin{array}{cc} \frac{1}{\tau_{\mathrm{tr}}^{\left(\lambda ,\xi \right)}\left(k_{F}^{\xi }\right)} & =\frac{1}{\tau^{\left(\lambda ,\xi \right)}\left(k_{F}^{\xi }\right)}-\frac{1}{\tau_{1}^{\left(\lambda ,\xi \right)}\left(k_{F}^{\xi }\right)}\\ \; & =\frac{2\pi n_{d}}{\hbar }\int \frac{d^{2}k^{\prime }}{{\left(2\pi \right)}^{2}}\delta \left(\hbar v_{F}k_{F}^{\xi }-\hbar v_{F}k^{\prime }\right){\left|T_{k_{\|}^{\prime }k_{\|}}^{\left(\lambda ,\xi \right)}\right|}^{2}\left(1-\cos\phi^{\prime }\right). \end{array}$$

Using the analytical expression for the *T*-matrix elements in Equation (16), we obtain a closed expression for the transport relaxation time in terms of the scattering phase shifts $\delta_{m}\left(k\right)$

(50) $$\frac{1}{\tau_{\mathrm{tr}}^{\left(\lambda ,\xi \right)}\left(k_{F}^{\xi }\right)}=\frac{2n_{d}v_{F}}{k_{F}^{\xi }}\sum_{m=-\infty }^{\infty }\sin^{2}\left[\delta_{m}\left(k_{F}^{\xi }\right)-\delta_{m-1}\left(k_{F}^{\xi }\right)\right].$$

From Equation (48), we can investigate the zero temperature T→0 and high temperature $T\gg \hbar v_{F}k_{F}/k_{B}$ limits, respectively. In the zero temperature limit, we obtain

(51) $$\sigma_{\alpha \alpha }^{\left(\lambda ,\xi \right)}\left(T\to 0\right)=\frac{2}{\pi^{2}}k_{F}^{\xi 2}\left(\frac{e^{2}}{\hbar }\right)v_{F,\alpha }\tau_{\mathrm{tr}}^{\left(\lambda ,\xi \right)}\left(k_{F}^{\xi }\right),$$

a constant that depends on the microscopic material properties (such as $v_{F}$), as well as on the concentration of dislocations $n_{d}$ through the relaxation time.

On the other hand, in the high-temperature limit $T\gg \hbar v_{F}k_{F}/k_{B}$, we obtain a quadratic dependence on temperature

(52) $$\sigma_{\alpha \alpha }^{\left(\lambda ,\xi \right)}\left(T\gg \hbar v_{F}k_{F}/k_{B}\right)=\frac{1}{3v_{F,\alpha }}\left(\frac{e^{2}}{\hbar }\right){\left(\frac{k_{B}T}{\hbar }\right)}^{2}\tau_{\mathrm{tr}}^{\left(\lambda ,\xi \right)}\left(k_{F}^{\xi }\right),$$

where the overall constant depends on the microscopic parameters for each material, as well as on the concentration of dislocations through the relaxation time.

## 4. Results

In this section, we apply the theory and analytical expressions obtained in the previous section to calculate the electrical conductivity of several materials in the family of transition metals’ monopnictides, i.e., TaAs, TaP, NbAs, and NbP. For an estimation of the concentration of defects $n_{d}$ in real crystal systems, Ref. [24] reports that the native concentration of dislocations in the lattice of the materials TiO_2_ and SrTiO_3_ varies in the range $n_{d}∼10^{5}$–$10^{7}\;{\mathrm{cm}}^{-2}$. These concentrations can be enhanced using different treatments up to $10^{13}\;{\mathrm{cm}}^{-2}$, close to the rendering amorphous limit. The microscopic/atomistic parameters involved in our theory are obtained from *ab-initio* studies for WSM materials, as reported in Refs. [25,26]. In particular, the later reference identifies anisotropies in the Fermi velocities and density of charge carries at different Weyl nodes and bands. Using these results for the densities of carriers, we compute the Fermi momentum at each Weyl node, i.e., $k_{F}^{\xi }$, as displayed in Table 1.

In what follows, for definiteness, we shall assume that the axis of the defects is along the crystallographic *z*-direction and that we are measuring the conductivity along the *x*-direction. Then, we use the reported *x*-components of the Fermi velocities [25,26]. We have different Fermi velocities $v_{F,x}^{\left(\lambda ,\xi \right)}$, for the conduction band (λ=+ 1) and for the valence band (λ=−1), and for each of the Weyl nodes (ξ=±), respectively. Actually, for the valence band, Refs. [25,26] report the *hole* velocity. Their results are presented in Table 2.

Now, in order to study the additional effect of the torsional dislocations, we follow our previous work [22] to estimate the geometrical parameters involved in the model. We assume that the dislocations are cylindrical regions along the *z*-axis with radius *a*. Here, we further assume that the defects possess an average radius of a=15 nm. The simple relation between the torsional angle θ (in degrees) and the pseudo-magnetic field representing strain is $B_{S}a^{2}=1.36\;\theta \;{\tilde{\phi }}_{0}$ [22], where the modified flux quantum in this material is approximately ${\tilde{\phi }}_{0}\equiv \frac{\hbar v_{F}}{e}=\frac{1}{2\pi }\frac{v_{F}}{c}\frac{hc}{e}=\frac{1}{2\pi }\frac{1.5}{300}\cdot 4.14\times 10^{5}T{\overset{˚}{A}}^{2}\approx 330\;T{\overset{˚}{A}}^{2}$. In this work, we have chosen a torsion angle $\theta =15^{\circ }$. The lattice mismatch effect at the surface of the dislocation cylinders is modeled by a repulsive delta-potential, with strength $V_{0}$, expressed in terms of the “spinor rotation” angle $\alpha =V_{0}/\hbar v_{F}$. According to our previous work [22], a realistic choice is α=3π/4.

With all of these parameters fixed, we can compute the transport relaxation time for each material from Equation (50). Our results are presented in Table 3.

Now, we compute the conductivity along the *x*-direction $\sigma_{xx}$. In what follows, we simply call it σ(T), as a function of temperature. The total conductivity is the sum over nodes and bands

(53) $$\sigma \left(T\right)=\sum_{\xi =\pm 1}\sum_{\lambda =\pm 1}\sigma_{xx}^{\left(\lambda ,\xi \right)}\left(T\right),$$

where $\sigma_{xx}^{\left(\lambda ,\xi \right)}\left(T\right)$ is given in Equation (48), including the vertex correction. Our results for T=0 are presented in Table 4.

**Figure 7:**
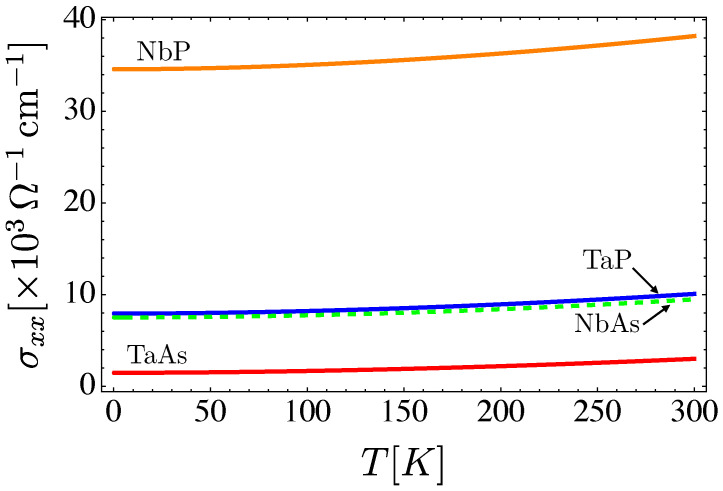
A comparison of the total conductivity versus temperature behavior for the transition metals’ monopnictides TaAs, TaP, NbAs and NbP. Here, we use a concentration of dislocations of $n_{d}=10^{11}\;{\mathrm{cm}}^{-2}$.

**Figure 8:**
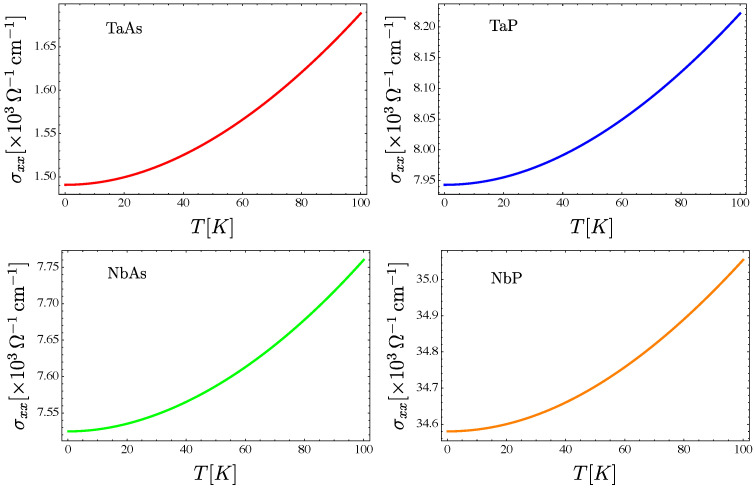
The total conductivity vs. temperature behavior for the transition metals monopnictides TaAs, TaP, NbAs, and NbP. Here, we use a concentration of dislocations of $n_{d}=10^{11}\;{\mathrm{cm}}^{-2}$.

The conductivity as a function of temperature, for the transition metals’ monopnictides TaAs, TaP, NbAs and NbP, is presented in Figure 7 for all of them compared, and individually in the panel Figure 8.

**Figure 9:**
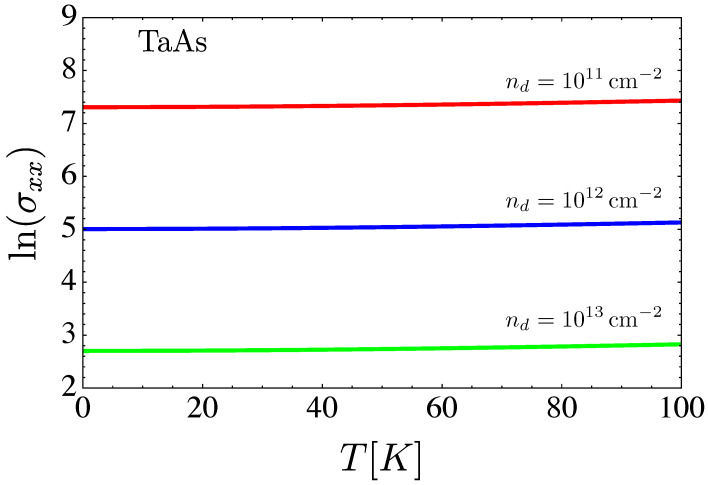
Natural logarithm of the conductivity versus temperature for three different concentrations of dislocations.

Now, let us study the conductivity behavior with respect to the density of dislocations $n_{d}$. In Figure 9, we present a plot of the natural logarithm of the conductivity versus temperature for three different concentrations of dislocations.

**Figure 10:**
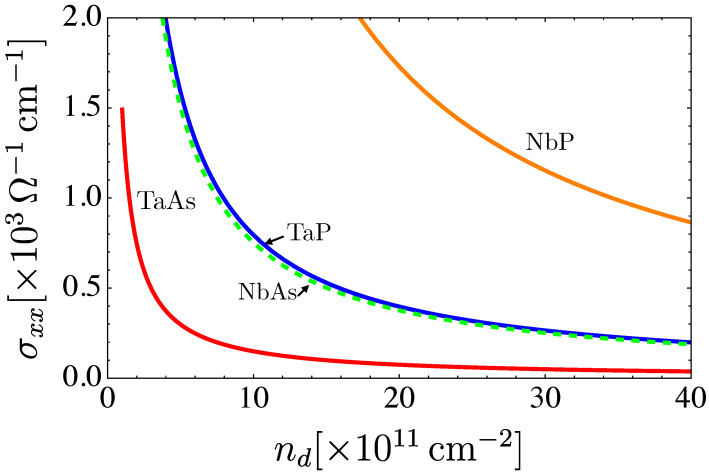
Plot of total conductivity versus defects’ concentration. The graphs were computed at zero temperature.

The total conductivity as a function of the concentration of defects and at zero temperature is presented in Figure 10.

**Figure 11:**
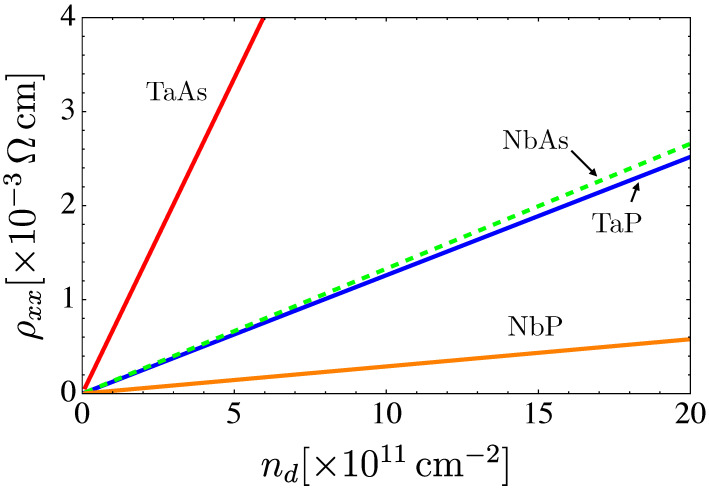
Total resistance, R=1/G, as a function of the concentration of defects $n_{d}$ for the family of materials TaAs, TaP, NbAs and NbP. The graphs were computed at zero temperature.

Finally, a plot of the resistance, defined as the inverse of conductivity, as a function of the dislocations’ density is presented in Figure 11.

## 5. Discussion and Conclusions

In this work, we have studied the effect of a distribution of mechanical defects, i.e., torsional dislocations, over the electrical conductivity of the family of transition metals monopnictides TaAs, TaP, NbAs and NbP. Our theory is based on the mathematical analysis of the scattering phase shifts from a single defect, as stated in our previous work [20,21,22,23,27]. We extended this previous analysis to develop a Green´s function formalism, in order to represent the scattering due to a finite concentration of randomly distributed defects. Within the non-crossing approximation for the self-energy, we solved explicitly for the disorder-averaged retarded Green´s function that allows us to calculate the electrical conductivity in the Kubo linear-response formalism. We obtained general analytical expressions in terms of the parameters involved in the low-energy model representing the family of materials, and using the *ab-initio* estimations for such parameters, we provided a characterization of the conductivity as a function of temperature and concentration of defects for the transition metal monopnictides TaAs, TaP, NbAs and NbP. As a universal feature, we identified a $∼T^{2}$ temperature dependence for $T\gg \hbar v_{F}k_{F}/k_{B}$, where the pre-factor depends on material-specific microscopic parameters as well as in the concentration of dislocations $n_{d}$ through the scattering relaxation time. Our results do not involve the electron–phonon scattering effects that will presumably contribute at finite temperatures. However, those can be included via Mathiessen’s rule in an overall relaxation time combining Equation (50) for $\tau_{tr}$ with a separate theoretical estimation for the electron–phonon relaxation time $\tau_{e-ph}$, as follows: $\tau^{-1}=\tau_{tr}^{-1}+\tau_{e-ph}^{-1}$. Since the electron–phonon interaction that determines the magnitude of $\tau_{e-ph}$ is an entirely different physical mechanism, it deserves an analysis on its own, to be communicated in a separate article which is under current development. 

## Figures and Tables

**Table 1:** Values of $k_{F}^{\xi }$ computed from the carrier densities reported in Ref. [26].

Material	$k_{F}^{+}[{\mathrm{nm}}^{-1}]$	$k_{F}^{-}[{\mathrm{nm}}^{-1}]$
TaAs	0.23	0.05
TaP	0.50	0.09
NbAs	0.46	0.03
NbP	1.04	0.15

**Table 2:** Values of the Fermi velocity $v_{F,x}^{\left(\lambda ,\xi \right)}$ in units of $10^{5}$ m/s, as reported in Ref. [26]. Notice that, for the valence bands (λ=−1), they report the hole velocity.

Material	$v_{F,x}^{\left(+,+\right)}$	$v_{F,x}^{\left(-,+\right)}$	$v_{F,x}^{\left(+,-\right)}$	$v_{F,x}^{\left(-,-\right)}$
TaAs	3.2	−5.3	2.6	−4.3
TaP	3.7	−5.4	2.0	−3.9
NbAs	3.0	−4.8	2.5	−3.2
NbP	3.0	−5.1	1.7	−2.4

**Table 3:** Computed values for the total relaxation time and the total transport relaxation time for each material after Equation (50). We consider a concentration of dislocations $n_{d}=10^{11}\;{\mathrm{cm}}^{-2}$.

Material	τ [$10^{-13}$ s]	$\tau_{\mathrm{tr}}$ [$10^{-13}$ s]
TaAs	2.2	2.6
TaP	2.4	3.2
NbAs	2.2	3.1
NbP	2.4	4.2

**Table 4:** Computed values for the total conductivity $\sigma_{0}=\sigma \left(T=0\right)$ at zero temperature for each material. We consider a value of $n_{d}=10^{11}\;{\mathrm{cm}}^{-2}$.

Material	$\sigma_{0}$ [$10^{3}$ $\Omega^{-1}\;{\mathrm{cm}}^{-1}$]
TaAs	1.5
TaP	7.9
NbAs	7.5
NbP	34.6

## Data Availability

Not applicable.

## Abbreviations

The following abbreviations are used in this manuscript:

WSM	Weyl semimetal
NCA	Non-crossing approximation

## Appendix A. Calculation of the Retarded Free Green’s Function

The free retarded Green’s function (GF) is represented in the coordinate basis as follows:

(A1) $$G_{R,0}^{\xi }\left(r,r^{\prime };E\right)=\langle r|\frac{1}{E-{\hat{H}}_{0}^{\xi }+i\eta^{+}}|r^{\prime }\rangle .$$

On this basis, $G_{R,0}^{\xi }\left(r,r^{\prime };E\right)$ satisfies the differential equation

(A2) $$\left(E+i\eta^{+}-\xi v_{F}\sigma \cdot \frac{\hbar }{i}\nabla \right)G_{R,0}^{\xi }\left(r,r^{\prime };E\right)=\sigma_{0}\delta^{\left(3\right)}\left(r-r^{\prime }\right).$$

where $\sigma_{0}$ is the 2×2 unit matrix. Let us introduce the scalar GF, $G_{R,0}^{\xi }\left(r,r^{\prime }\right)$, by means of the expression

(A3) $$G_{R,0}^{\xi }\left(r,r^{\prime };E\right)=\left(E+i\eta^{+}+\xi v_{F}\sigma \cdot \frac{\hbar }{i}\nabla \right)G_{R,0}^{\xi }\left(r,r^{\prime }\right).$$

Bearing in mind that we are treating the elastic scattering problem, the energy of the out-state must be the same as those of the incident-free-particle state, i.e., $E=\lambda \xi \hbar v_{F}\left|k\right|$. Then, the scalar GF satisfies the Helmholtz equation

(A4) $$\left(\nabla^{2}+k^{2}+i\eta^{+}\right)G_{R,0}^{\xi }\left(r,r^{\prime }\right)=\frac{1}{\hbar^{2}v_{F}^{2}}\delta^{\left(3\right)}\left(r-r^{\prime }\right).$$

Due to the symmetry along the *z*-axis, we can decouple it from its perpendicular plane as follows:

(A5) $$G_{R,0}^{\xi }\left(r,r^{\prime };k\right)=\int_{-\infty }^{\infty }\frac{dq_{z}}{2\pi }e^{iq_{z}\left(z-z^{\prime }\right)}G_{R,0}^{\xi }\left(x,x^{\prime };q_{z},k\right),$$

where $G_{R,0}^{\xi }\left(x,x^{\prime };q_{z},k\right)$ is a reduced GF and x=(x,y) is the position vector on the plane. Then, the Helmholtz equation for the reduced GF on the plane takes the form

(A6) $$\left(\nabla_{\|}^{2}-q_{z}^{2}+k^{2}+i\eta^{+}\right)G_{R,0}^{\xi }\left(x,x^{\prime };q_{z},k\right)=\frac{1}{\hbar^{2}v_{F}^{2}}\delta^{\left(2\right)}\left(x-x^{\prime }\right),$$

where $\nabla_{\|}^{2}=\partial_{x}^{2}+\partial_{y}^{2}$. As can be seen from the Figure 1, the free incident particle’s propagation is normal to the cylinder’s axis. We assume that the incident particles have negligible momentum along the *z*-axis, and by momentum conservation, they remain with negligible momentum along that direction during the transport process. Then, we can write $k=(k_{\|},0)$ where $k_{\|}=\left(k_{x},k_{y}\right)$. Hence, the system is reduced to an effective two-dimensional description, and we can consider the reduced GF on the plane as independent of the Fourier mode $q_{z}$. Then, from Equation (A5), we have $G_{R,0}^{\xi }\left(r,r^{\prime };k\right)=\delta \left(z-z^{\prime }\right)G_{R,0}^{\xi }\left(x,x^{\prime };k\right)$, and we can expand the reduced GF on the plane in the traverse Fourier space

(A7) $$G_{R,0}^{\xi }\left(x,x^{\prime };k\right)=\int \frac{d^{2}q_{\|}}{{\left(2\pi \right)}^{2}}e^{iq_{\|}\cdot \left(x-x^{\prime }\right)}{\tilde{G}}_{R,0}^{\xi }\left(q_{\|}\right),$$

where $q_{\|}=\left(q_{x},q_{y}\right)$. Replacing in Equation (A6), we obtain in the traverse Fourier space

(A8) $${\tilde{G}}_{R,0}^{\xi }\left(q_{\|}\right)=-\frac{1}{\hbar^{2}v_{F}^{2}}\frac{1}{q_{\|}^{2}-k^{2}-i\eta^{+}}.$$

We perform the integration in Equation (A7) in polar coordinates

(A9) $$\begin{array}{cc} G_{R,0}^{\xi }\left(x,x^{\prime };k\right)\; & =-\frac{1}{\hbar^{2}v_{F}^{2}}\frac{2\pi }{{\left(2\pi \right)}^{2}}\int_{0}^{\infty }dq_{\|}\;q_{\|}\frac{J_{0}\left(q_{\|}R\right)}{q_{\|}^{2}-k^{2}-i\eta^{+}}, \end{array}$$

where $R=|x-x^{\prime }|$, and we have used the integral representation of the Bessel functions $\int_{0}^{2\pi }e^{iz\cos\phi \pm \sin\phi }d\phi =2\pi i^{n}J_{n}\left(z\right)$. In order to perform the last integration, we need the result

(A10) $$\int_{0}^{\infty }\frac{x^{\nu +1}J_{\nu }\left(bx\right)}{{\left(x^{2}+a^{2}\right)}^{\mu +1}}dx=\frac{a^{\nu -\mu }b^{\mu }}{2^{\mu }\Gamma \left(\mu +1\right)}K_{\nu -\mu }\left(ab\right),$$

together with the relation $K_{n}\left(z\right)=\frac{\pi }{2}i^{n+1}H_{n}^{\left(1\right)}\left(iz\right)$. The result is

(A11) $$G_{R,0}^{\xi }\left(x,x^{\prime };k\right)=-\frac{i}{4\hbar^{2}v_{F}^{2}}H_{0}^{\left(1\right)}\left(k|x-x^{\prime }|\right).$$

This form is adequate because, in the asymptotic form for large $|x-x^{\prime }|$, it produces outgoing cylindrical waves as is desired for the retarded GF. Now, to obtain the final form for the free GF matrix, we apply the definition in Equation (A3) with $E=\lambda \xi \hbar v_{F}\left|k\right|$, taking into account that we have reduced to a two-dimensional system on the plane *x*-*y*

(A12) $$\begin{array}{c} G_{R,0}^{\xi }\left(r,r^{\prime };k\right)=-\frac{i\lambda \xi }{4\hbar v_{F}}\delta \left(z-z^{\prime }\right)\left(k\sigma_{0}-i\lambda \sigma \cdot \nabla_{\|}\right)H_{0}^{\left(1\right)}\left(k|x-x^{\prime }|\right). \end{array}$$

In plane polar coordinates,

(A13) $$\sigma \cdot \nabla_{\|}=\left(\sigma \cdot \hat{r}\right)\frac{\partial }{\partial r}+\left(\sigma \cdot \hat{\varphi }\right)\frac{1}{r}\frac{\partial }{\partial \varphi }$$

where $r=|x-x^{\prime }|$, φ is the angle the vector $x-x^{\prime }$ makes with the *x* axis and

(A14) $$\sigma \cdot \hat{r}=\left(\begin{array}{cc} 0 & e^{-i\varphi }\\ e^{i\varphi } & 0 \end{array}\right),\;\sigma \cdot \hat{\varphi }=\left(\begin{array}{cc} 0 & -ie^{-i\varphi }\\ ie^{i\varphi } & 0 \end{array}\right).$$

The final form for the retarded Green’s function matrix in the coordinates representation is

(A15) $$\begin{array}{c} G_{R,0}^{\xi }\left(r,r^{\prime };k\right)=-\frac{\lambda \xi ik}{4\hbar v_{F}}\delta \left(z-z^{\prime }\right)\left[\begin{array}{cc} H_{0}^{\left(1\right)}\left(k|x-x^{\prime }|\right) & i\lambda e^{-i\varphi }H_{1}^{\left(1\right)}\left(k|x-x^{\prime }|\right)\\ i\lambda e^{i\varphi }H_{1}^{\left(1\right)}\left(k|x-x^{\prime }|\right) & H_{0}^{\left(1\right)}\left(k|x-x^{\prime }|\right) \end{array}\right], \end{array}$$

which produces Equation (9).

## Appendix B. Scattering by a Single Cylindrical Defect

We can represent the Lippmann–Schwinger Equation (7) in the coordinate basis as follows:

(A16) $$\begin{array}{c} \Psi_{k,\lambda }\left(r\right)=\Phi_{k,\lambda }\left(r\right)+\int d^{3}r^{\prime }\int d^{3}r^{\prime \prime }\langle r|{\hat{G}}_{R,0}^{\xi }\left(E\right)|r^{\prime }\rangle \langle r^{\prime }\left|{\hat{T}}^{\xi }\left(E\right)\right|r^{\prime \prime }\rangle \Phi_{k,\lambda }\left(r^{\prime \prime }\right). \end{array}$$

The form of free spinors in Equation (6) with momentum $k_{\|}$ on the *x*–*y* plane is

(A17) $$\Phi_{\lambda ,k}\left(r\right)=\frac{1}{\sqrt{2}}\left(\begin{array}{c} 1\\ \lambda e^{i\phi } \end{array}\right)e^{ik\cdot r}\equiv \frac{1}{\sqrt{2}}\left(\begin{array}{c} 1\\ \lambda e^{i\phi } \end{array}\right)e^{ik_{\|}\cdot x}=\Phi_{\lambda ,k_{\|}}\left(x\right).$$

where $k_{x}=k\cos\phi$ and $k_{y}=k\sin\phi$. The incident spinors are assumed to enter the scattering region with momentum along the *x*-axis, and they are represented by

(A18) $$\Phi_{\lambda ,k_{\|}}^{\mathrm{inc}}\left(x\right)=\frac{1}{\sqrt{2}}\left(\begin{array}{c} 1\\ \lambda \end{array}\right)e^{ikx}.$$

Now, ${\hat{H}}_{1}^{\xi }$ is a local potential independent of the *z* coordinate as can be seen from Equation (). Then, the *T*-matrix is diagonal in the coordinate basis and depends only on vectors x on the plane. Thus, $\langle r^{\prime }\left|{\hat{T}}^{\xi }\left(E\right)\right|r^{\prime \prime }\rangle =T^{\xi }\left(x^{\prime },E\right)\delta^{\left(3\right)}\left(r^{\prime }-r^{\prime \prime }\right)$, where $T^{\xi }\left(x^{\prime },E\right)$ is a 2×2 matrix. The incident spinor is given in Equation (A18), and using the retarded GF in Equation (A15), the Lippmann–Schwinger equation in Equation (A16) is reduced to the *x*–*y* plane as follows:

(A19) $$\begin{array}{c} \Psi_{k_{\|},\lambda }\left(x\right)=\frac{1}{\sqrt{2}}\left(\begin{array}{c} 1\\ \lambda \end{array}\right)e^{ikx}+\int d^{2}x^{\prime }G_{R,0}^{\xi }\left(x,x^{\prime };k\right)T^{\xi }\left(x^{\prime },E\right)\Phi_{k_{\|},\lambda }\left(x^{\prime }\right), \end{array}$$

where $G_{R,0}^{\xi }\left(x,x^{\prime };k\right)$ is given in Equation (9). The asymptotic form for large argument of the Hankel’s functions are

(A20) $$\begin{array}{cc} H_{0}^{\left(1\right)}(k|x-x^{\prime }\left|\right) & ∼\sqrt{\frac{2}{i\pi k|x-x^{\prime }|}}e^{ik|x-x^{\prime }|}, \end{array}$$

(A21) $$\begin{array}{cc} H_{1}^{\left(1\right)}(k|x-x^{\prime }\left|\right) & ∼-i\sqrt{\frac{2}{i\pi k|x-x^{\prime }|}}e^{ik|x-x^{\prime }|}, \end{array}$$

where we have used the known limiting form

(A22) $$H_{\nu }^{\left(1\right)}\left(x\right)∼\sqrt{\frac{2}{\pi x}}e^{i\left(x-\frac{\nu \pi }{2}-\frac{\pi }{4}\right)},\;x\to \infty .$$

Now, recall the geometry of the scattering process as depicted in Figure 2. We expand $|x-x^{\prime }|$ for large |x| as follows:

(A23) $$|x-x^{\prime }|∼r-\hat{n}\cdot x^{\prime }+O\left({\left(r^{\prime }/r\right)}^{2}\right),$$

where r=|x|, $r^{\prime }=\left|x^{\prime }\right|$ and $\hat{n}$ is the unit vector in the direction of x, i.e., $\hat{n}=x/r$. Noting that in this asymptotic form the direction of $k_{\|}^{\prime }$ coincides with that of x and is practically the same of $x-x^{\prime }$, i.e., $k_{\|}^{\prime }=k\hat{n}$ and that the angle ϕ and the vector $k_{\|}^{\prime }$ make the $k_{\|}$ incident momentum be approximately the same angle $x-x^{\prime }$, i.e., ϕ∼φ, we have the asymptotic form for the free Green’s function in Equation (9)

(A24) $$G_{R,0}^{\xi }\left(x,x^{\prime };k\right)∼-\frac{\lambda \xi k}{4\hbar v_{F}}\sqrt{\frac{2i}{\pi k}}\left[\begin{array}{cc} 1 & \lambda e^{-i\phi }\\ \lambda e^{i\phi } & 1 \end{array}\right]e^{-ik_{\|}^{\prime }\cdot x^{\prime }}\frac{e^{ikr}}{\sqrt{r}}.$$

Replacing the asymptotic form in Equation (A24) in Equation (A19), we obtain Equation (14)

(A25) $$\Psi_{k_{\|},\lambda }\left(x\right)∼\frac{1}{\sqrt{2}}\left(\begin{array}{c} 1\\ \lambda \end{array}\right)e^{ikx}-\frac{\lambda \xi }{2\hbar v_{F}}\sqrt{\frac{ik}{\pi }}T_{k_{\|}^{\prime }k_{\|}}^{\left(\lambda ,\xi \right)}\left(\begin{array}{c} 1\\ \lambda e^{i\phi } \end{array}\right)\frac{e^{ikr}}{\sqrt{r}},$$

where the *T*-matrix elements are

(A26) $$T_{k_{\|}^{\prime }k_{\|}}^{\left(\lambda ,\xi \right)}\left(E\right)=\int d^{2}x^{\prime }\Phi_{k_{\|}^{\prime },\lambda }^{†}\left(x^{\prime }\right)T^{\xi }\left(x^{\prime },E\right)\Phi_{k_{\|},\lambda }\left(x^{\prime }\right).$$

In order to compute the *T*-matrix elements, we need the phase shifts, whose analytical expression is presented in Equation (32) of the supplemental material of our previous work [22]. Here, we reproduce the final result

(A27) $$\tan\delta_{m}=\frac{\beta J_{m+1}-\varrho_{n}^{\xi }J_{m}\cdot z_{a}^{\frac{\left|m+1|-|m\right|}{2}}\frac{L_{n_{\rho }^{\prime }}^{\left|m+1\right|}\left(z_{a}\right)}{L_{n_{\rho }}^{\left|m\right|}\left(z_{a}\right)}+\tan\alpha \left[J_{m}+\beta \varrho_{n}^{\xi }J_{m+1}\cdot z_{a}^{\frac{\left|m+1|-|m\right|}{2}}\frac{L_{n_{\rho }^{\prime }}^{\left|m+1\right|}\left(z_{a}\right)}{L_{n_{\rho }}^{\left|m\right|}\left(z_{a}\right)}\right]}{\beta Y_{m+1}-\varrho_{n}^{\xi }Y_{m}\cdot z_{a}^{\frac{\left|m+1|-|m\right|}{2}}\frac{L_{n_{\rho }^{\prime }}^{\left|m+1\right|}\left(z_{a}\right)}{L_{n_{\rho }}^{\left|m\right|}\left(z_{a}\right)}+\tan\alpha \left[Y_{m}+\beta \varrho_{n}^{\xi }Y_{m+1}\cdot z_{a}^{\frac{\left|m+1|-|m\right|}{2}}\frac{L_{n_{\rho }^{\prime }}^{\left|m+1\right|}\left(z_{a}\right)}{L_{n_{\rho }}^{\left|m\right|}\left(z_{a}\right)}\right]},$$

where $\delta_{m}$ is a function of *k* through the Bessel functions $J_{m}\equiv J_{m}\left(ka\right)$ and $Y_{m}\equiv Y_{m}\left(ka\right)$, and $z_{a}=\left|B_{\xi }\right|a^{2}/2{\tilde{\phi }}_{0}$ (*a* is the cylinder’s radius).

## Appendix C. The Spectral Function

The spectral function can be defined as follows:

(A28) $${\hat{A}}^{\xi }\left(E\right)=i\left[{\hat{G}}_{R}^{\xi }\left(E\right)-{\hat{G}}_{A}^{\xi }\left(E\right)\right],$$

in terms of the complete retarded and advanced Green’s functions. Then, the spectral function is Hermitian ${\left[{\hat{A}}^{\xi }\left(E\right)\right]}^{†}={\hat{A}}^{\xi }\left(E\right)$. Given the averaged complete retarded Green’s function in Equation (26), the form of the spectral function in momentum space is

(A29) $$\begin{array}{cc} A^{\lambda ,\xi }\left(k_{\|}\right) & =i\left[\langle G_{R}^{\lambda ,\xi }\left(k_{\|}\right)\rangle -\langle G_{A}^{\lambda ,\xi }\left(k_{\|}\right)\rangle \right]\\ \; & =i\left[\frac{1}{E-\lambda \xi \hbar v_{F}k-\Sigma_{R}^{\lambda ,\xi }\left(k_{\|}\right)}-\frac{1}{E-\lambda \xi \hbar v_{F}k-\Sigma_{A}^{\lambda ,\xi }\left(k_{\|}\right)}\right]. \end{array}$$

Clearly, it takes the form of a Lorentzian distribution with compact support around the free particle’s energy

(A30) $$A^{\lambda ,\xi }\left(k\right)=\frac{2\left(\frac{\hbar }{2\tau^{\left(\lambda ,\xi \right)}\left(k\right)}\right)}{{\left(E-E_{k}^{\lambda ,\xi }\right)}^{2}+{\left(\frac{\hbar }{2\tau^{\left(\lambda ,\xi \right)}\left(k\right)}\right)}^{2}},$$

where $\tau^{\left(\lambda ,\xi \right)}\left(k\right)$ is the *relaxation time* and $E_{k}^{\lambda ,\xi }=\lambda \xi \hbar v_{F}k$. In the limit of low concentration of defects, i.e., large relaxation time because of Equation (31), the spectral function becomes a delta distribution

(A31) $$\lim_{\tau \to \infty }A^{\lambda ,\xi }\left(k\right)=2\pi \delta \left(E-E_{k}^{\lambda ,\xi }\right).$$

Due to its behavior as a Lorentzian, the spectral function has the important property [29]

(A32) $$A^{\lambda ,\xi }\left(k\right)A^{\lambda^{\prime },\xi }\left(k^{\prime }\right)=A^{\lambda ,\xi }\left(k\right)A^{\lambda ,\xi }\left(k\right)\delta_{\lambda \lambda^{\prime }}.$$

Representing the spectral function Equation (A28) in the coordinate basis using the complete set of eigenstates of the full Hamiltonian, we have

(A33) $${𝓐}^{\xi }\left(r,r^{\prime }\right)=\int \frac{d^{3}k}{{\left(2\pi \right)}^{3}}e^{ik\cdot (r-r^{\prime })}\sum_{\lambda }\left(\sigma_{0}+\lambda \frac{\sigma \cdot k_{\|}}{|k_{\|}|}\right)A^{\lambda ,\xi }\left(k\right).$$

Notice that, because $k_{\|}=\left(k_{x},k_{y}\right)$, when we perform the integration, the spectral function takes the form ${𝓐}^{\xi }\left(r,r^{\prime }\right)=\delta \left(z-z^{\prime }\right){𝓐}^{\xi }\left(x,x^{\prime }\right)$, which looks similar to the decoupled form of the GF in Equation (A15).

## Appendix D. Linear Response Theory

The tensor $K_{\alpha \beta }$ is defined using the retarded current–current correlator in Equation (35). Introducing in Equation (35) the complete and orthonormal basis $\left\{|\Psi_{\lambda ,k}\rangle \right\}$ of the total Hamiltonian, such that ${\hat{H}}^{\xi }|\Psi_{\lambda ,k}\rangle =E_{k}^{\lambda ,\xi }|\Psi_{\lambda ,k}\rangle$ and $\hat{\rho }|\Psi_{\lambda ,k}\rangle =\rho \left(E_{k}^{\lambda ,\xi }\right)|\Psi_{\lambda ,k}\rangle$, we obtain

(A34) $$\begin{array}{cc} K_{\alpha \beta }^{\xi }\left(r,t;r^{\prime },t^{\prime }\right)= & i\hbar^{-1}\theta \left(t-t^{\prime }\right)\int \frac{d^{3}k}{{\left(2\pi \right)}^{3}}\int \frac{d^{3}k^{\prime }}{{\left(2\pi \right)}^{3}}\sum_{\lambda ,\lambda^{\prime }}\left[\rho \left(E_{k}^{\lambda ,\xi }\right)-\rho \left(E_{k^{\prime }}^{\lambda^{\prime },\xi }\right)\right]\\ \; & \;\times \langle \Psi_{\lambda ,k}\left|{\hat{j}}_{\alpha }\left(r\right)\right|\Psi_{\lambda^{\prime },k^{\prime }}\rangle \langle \Psi_{\lambda^{\prime },k^{\prime }}\left|{\hat{j}}_{\beta }\left(r^{\prime }\right)\right|\Psi_{\lambda ,k}\rangle e^{\frac{i}{\hbar }\left(E_{k}^{\lambda ,\xi }-E_{k^{\prime }}^{\lambda^{\prime },\xi }\right)\left(t-t^{\prime }\right)}. \end{array}$$

Using the Fourier representation of the Heaviside step function, we obtain the correlator in the frequency domain

(A35) $$\begin{array}{cc} K_{\alpha \beta }^{\xi }\left(r,r^{\prime };\omega \right)= & \int \frac{d^{3}k}{{\left(2\pi \right)}^{3}}\int \frac{d^{3}k^{\prime }}{{\left(2\pi \right)}^{3}}\sum_{\lambda ,\lambda^{\prime }}\frac{\left[\rho \left(E_{k^{\prime }}^{\lambda^{\prime },\xi }\right)-\rho \left(E_{k}^{\lambda ,\xi }\right)\right]}{\hbar \omega +E_{k}^{\lambda ,\xi }-E_{k^{\prime }}^{\lambda^{\prime },\xi }+i\eta^{+}}\\ \; & \times \langle \Psi_{\lambda ,k}\left|{\hat{j}}_{\alpha }\left(r\right)\right|\Psi_{\lambda^{\prime },k^{\prime }}\rangle \langle \Psi_{\lambda^{\prime },k^{\prime }}\left|{\hat{j}}_{\beta }\left(r^{\prime }\right)\right|\Psi_{\lambda ,k}\rangle . \end{array}$$

The electric current density operator for the Weyl equation is ${\hat{j}}^{\xi }\left(r\right)=-e\xi v_{F}|r\rangle \sigma \langle r|$. Then,

(A36) Kαβξ(r,r′;ω)=e2vF2∫d3k(2π)3∫d3k′(2π)3∑λ,λ′ρEk′λ′,ξ−ρEkλ,ξℏω+Ekλ,ξ−Ek′λ′,ξ+iη+=×TrσαΨλ′,k′(r)⊗Ψλ′,k′†(r′)σβΨλ,k(r′)⊗Ψλ,k†(r).

We can rewrite this last expression in terms of the spectral function and the retarded/advanced GFs as follows:

(A37) $$\begin{array}{cc} K_{\alpha \beta }^{\xi }\left(r,r^{\prime };\omega \right)= & -2e^{2}v_{F}^{2}\int_{-\infty }^{\infty }\frac{dE}{2\pi }f_{0}\left(E\right)\mathrm{Tr}\left[\sigma_{\alpha }{𝓐}^{\xi }\left(r,r^{\prime };E\right)\sigma_{\beta }G_{A}^{\xi }\left(r^{\prime },r;E-\hbar \omega \right)\right.\\ \; & \;\left.+\sigma_{\alpha }G_{R}^{\xi }\left(r,r^{\prime };E+\hbar \omega \right)\sigma_{\beta }{𝓐}^{\xi }\left(r^{\prime },r;E\right)\right], \end{array}$$

where the additional factor of 2 is due to the spin degeneracy, and $f_{0}\left(E\right)$ is the Fermi distribution. In the first term, we can shift energy variable E→E+ℏω, such that

(A38) $$\begin{array}{cc} K_{\alpha \beta }^{\xi }\left(r,r^{\prime };\omega \right)= & -2e^{2}v_{F}^{2}\int_{-\infty }^{\infty }\frac{dE}{2\pi }\left[f_{0}\left(E+\hbar \omega \right)\mathrm{Tr}\;\sigma_{\alpha }{𝓐}^{\xi }\left(r,r^{\prime };E+\hbar \omega \right)\sigma_{\beta }G_{A}^{\xi }\left(r^{\prime },r;E\right)\right.\\ \; & \;\;\;\left.+f_{0}\left(E\right)\mathrm{Tr}\;\sigma_{\alpha }G_{R}^{\xi }\left(r,r^{\prime };E+\hbar \omega \right)\sigma_{\beta }{𝓐}^{\xi }\left(r^{\prime },r;E\right)\right]. \end{array}$$

We are interested in the real part of the conductivity tensor. Then,

(A39) $$\begin{array}{cc} ℜ\;\sigma_{\alpha \beta }^{\xi }\left(r,r^{\prime };\omega \right) & =ℜ\;\left(\frac{1}{i\omega }K_{\alpha \beta }^{\xi }\left(r,r^{\prime };\omega \right)\right)\\ \; & =-\frac{i}{2\omega }\left[K_{\alpha \beta }^{\xi }\left(r,r^{\prime };\omega \right)-K_{\beta \alpha }^{\xi †}\left(r,r^{\prime };\omega \right)\right], \end{array}$$

where we have used the fact that $K_{\alpha \beta }^{*}=K_{\beta \alpha }^{†}$. Then, taking the Hermitian conjugate of Equation (A38) and using the cyclic property of the trace, we can write

(A40) $$\begin{array}{cc} ℜ\;\sigma_{\alpha \beta }^{\xi }\left(r,r^{\prime };\omega \right)= & -\frac{e^{2}\hbar v_{F}^{2}}{2\pi }\int_{-\infty }^{\infty }dE\left\{\frac{f_{0}\left(E+\hbar \omega \right)-f_{0}\left(E\right)}{\hbar \omega }\right\}\\ \; & \;\times \mathrm{Tr}\sigma_{\alpha }{𝓐}^{\xi }\left(r,r^{\prime };E+\hbar \omega \right)\sigma_{\beta }{𝓐}^{\xi }\left(r^{\prime },r;E\right). \end{array}$$

Introducing the spectral density in Equation (A33) in the DC conductivity expression in Equation (A40), we have

(A41) ℜσαβξ(r,r′;ω)=∫d3q(2π)3eiq·(r−r′)−e2ℏvF22π∫d3k′(2π)3∫−∞∞dEf0(E+ℏω)−f0(E)ℏω×∑λ,λ′Trσασ0+λσ·(k‖+q)|k‖+q|σβσ0+λ′σ·k‖|k‖|−e2ℏvF22π×Aλ,ξ(|k‖+q|;E+ℏω)Aλ′,ξ(|k‖|;E).

Then, the Fourier transform to the momentum space of the conductivity is the result in Equation (39). We are computing the DC conductivity, so we take the limit q→0 first and then the limit ω→0. The result is

(A42) $$\begin{array}{cc} \sigma_{\alpha \beta }^{\xi }\left(T\right)= & -\frac{e^{2}\hbar v_{F}^{2}}{2\pi }\int \frac{d^{3}k}{{\left(2\pi \right)}^{3}}\int_{-\infty }^{\infty }dE\frac{\partial f_{0}\left(E\right)}{E}\\ \; & \;\times \sum_{\lambda }\mathrm{Tr}\left\{\sigma_{\alpha }\left(\sigma_{0}+\lambda \frac{\sigma \cdot k_{\|}}{|k_{\|}|}\right)\sigma_{\beta }\left(\sigma_{0}+\lambda \frac{\sigma \cdot k_{\|}}{|k_{\|}|}\right)\right\}A^{\lambda ,\xi }\left(\right|k_{\|}\left|;E\right)A^{\lambda ,\xi }\left(\right|k_{\|}\left|;E\right). \end{array}$$

where we have used Equation (A32). Now, we perform the trace

(A43) $$\begin{array}{cc} \; & \mathrm{Tr}\left\{\sigma_{\alpha }\left(\sigma_{0}+\lambda \frac{\sigma \cdot k_{\|}}{|k_{\|}|}\right)\sigma_{\beta }\left(\sigma_{0}+\lambda \frac{\sigma \cdot k_{\|}}{|k_{\|}|}\right)\right\}\\ \; & \;=\mathrm{Tr}\left\{\sigma_{\alpha }\sigma_{\beta }+\lambda \left(\sigma_{\alpha }\sigma_{\beta }\sigma_{\gamma }+\sigma_{\alpha }\sigma_{\gamma }\sigma_{\beta }\right)\frac{k_{\gamma }}{|k_{\|}|}+\lambda^{2}\sigma_{\alpha }\sigma_{\gamma }\sigma_{\beta }\sigma_{\gamma^{\prime }}\frac{k_{\gamma }k_{\gamma^{\prime }}}{|k_{\|}{|}^{2}}\right\}\\ \; & \;=4\frac{k_{\alpha }k_{\beta }}{|k_{\|}{|}^{2}}, \end{array}$$

where we have used the Pauli matrices trace technology and the fact that $\lambda^{2}=1$. The angular integration is performed immediately as follows:

(A44) $$\int d\Omega \;\;k_{\alpha }k_{\beta }=k^{2}\int_{0}^{\pi }d\theta \sin\theta \int_{0}^{2\pi }d\phi \;\;n_{\alpha }n_{\beta }=2\pi k^{2}\delta_{\alpha \beta },$$

where $n_{\alpha }$ is the component of the unit vector n along the α-direction (on the plane) and $k_{\|}=kn$. Then, we have

(A45) $$\begin{array}{cc} \sigma_{\alpha \beta }^{\xi }\left(T\right) & =-\delta_{\alpha \beta }\frac{4e^{2}\hbar v_{F}^{2}}{{\left(2\pi \right)}^{3}k_{B}T}\sum_{\lambda }\int_{-\infty }^{\infty }dE\;\;f_{0}\left(E\right)\left[1-f_{0}\left(E\right)\right]\\ \; & \;\times \int_{0}^{\infty }dk\left[\langle G_{R}^{\lambda ,\xi }\left(k_{\|}\right)\rangle -\langle G_{A}^{\lambda ,\xi }\left(k_{\|}\right)\rangle \right]\left[\langle G_{R}^{\lambda ,\xi }\left(k_{\|}\right)\rangle -\langle G_{A}^{\lambda ,\xi }\left(k_{\|}\right)\rangle \right]k_{\|}\cdot k_{\|}, \end{array}$$

where we have used the definition of the spectral function in terms of the retarded and advanced GFs. In the limit of a low concentration of defects, i.e., $n_{d}\to 0$, we have that the unique leading contribution to the conductivity is given by the Lorentzian distribution

(A46) $$\begin{array}{cc} \langle G_{R}^{\lambda ,\xi }\left(k_{\|}\right)\rangle \langle G_{A}^{\lambda ,\xi }\left(k_{\|}\right)\rangle & =\frac{1}{{\left(E-\lambda \xi \hbar v_{F}k\right)}^{2}+{\left(\frac{\hbar }{2\tau^{\left(\lambda ,\xi \right)}\left(k\right)}\right)}^{2}}\\ \; & \;\to \frac{2\pi \tau^{\left(\lambda ,\xi \right)}\left(k\right)}{\hbar }\delta \left(E-\lambda \xi \hbar v_{F}k\right), \end{array}$$

where we have used the fact that the self-energy is purely imaginary and its relation with the relaxation time. The other contributions are negligible because they are not singular in $n_{d}$. For instance, in the low concentration limit, we have a contribution for two retarded GFs of the form

(A47) $$\begin{array}{cc} \langle G_{R}^{\lambda ,\xi }\left(k_{\|}\right)\rangle \langle G_{R}^{\lambda ,\xi }\left(k_{\|}\right)\rangle & =\frac{{\left(E-\lambda \xi \hbar v_{F}k\right)}^{2}-{\left(\frac{\hbar }{2\tau^{\left(\lambda ,\xi \right)}\left(k\right)}\right)}^{2}}{{\left\{{\left(E-\lambda \xi \hbar v_{F}k\right)}^{2}+{\left(\frac{\hbar }{2\tau^{\left(\lambda ,\xi \right)}\left(k\right)}\right)}^{2}\right\}}^{2}}\\ \; & \;+i\left(E-\lambda \xi \hbar v_{F}k\right)2\pi \delta \left(E-\lambda \xi \hbar v_{F}k\right). \end{array}$$

The second term is zero and the first term is a function centered at $E-\lambda \xi \hbar v_{F}k$, but when integrated over *k*, it gives zero. Something similar occurs with the contribution of two advanced GFs. The result is the diagonal conductivity tensor given in Equation (40).
